# A real-world longitudinal study of anemia management in non-dialysis-dependent chronic kidney disease patients: a multinational analysis of CKDopps

**DOI:** 10.1038/s41598-020-79254-6

**Published:** 2021-01-19

**Authors:** Marcelo Barreto Lopes, Charlotte Tu, Jarcy Zee, Murilo Guedes, Ronald L. Pisoni, Bruce M. Robinson, Bryce Foote, Katarina Hedman, Glen James, Antonio Alberto Lopes, Ziad Massy, Helmut Reichel, James Sloand, Sandra Waechter, Michelle M. Y. Wong, Roberto Pecoits-Filho

**Affiliations:** 1 Arbor Research Collaborative for Health, 3700 Earhart Road, Ann Arbor, MI 48105 USA; 2 Department of Internal Medicine, University of Michigan, Ann Arbor, MI USA; 3 Keryx Biopharmaceuticals, Inc, Boston, MA USA; 4 AstraZeneca, Gothenburg, Sweden; 5 AstraZeneca, Cambridge, UK; 6 Department of Internal Medicine, Federal University of Bahia, Salvador, Brazil; 7 Centre for Research in Epidemiology and Population Health (CESP), UMRS 1018, UVSQ, University Paris-Saclay, Villejuif, France; 8 Department of Nephrology, Ambroise Paré University Hospital, APHP, Boulogne Billancourt, Paris, France; 9 Nephrological Center, Villingen Schwenningen, Germany; 10 Vifor Pharma, Glattbrugg, Switzerland; 11 Department of Medicine, University of British Columbia, Vancouver, BC Canada; 12 School of Medicine, Pontificia Universidade Catolica Do Parana, Curitiba, Brazil

**Keywords:** Kidney diseases, Chronic kidney disease

## Abstract

Previously lacking in the literature, we describe longitudinal patterns of anemia prescriptions for non-dialysis-dependent chronic kidney disease (NDD-CKD) patients under nephrologist care. We analyzed data from 2818 Stage 3-5 NDD-CKD patients from Brazil, Germany, and the US, naïve to anemia medications (oral iron, intravenous [IV] iron, or erythropoiesis stimulating agent [ESA]) at enrollment in the CKDopps. We report the cumulative incidence function (CIF) of medication initiation stratified by baseline characteristics. Even in patients with hemoglobin (Hb) < 10 g/dL, the CIF at 12 months for any anemia medication was 40%, and 28% for ESAs. Patients with TSAT < 20% had a CIF of 26% and 6% for oral and IV iron, respectively. Heart failure was associated with earlier initiation of anemia medications. IV iron was prescribed to < 10% of patients with iron deficiency. Only 40% of patients with Hb < 10 g/dL received any anemia medication within a year. Discontinuation of anemia treatment was very common. Anemia treatment is initiated in a limited number of NDD-CKD patients, even in those with guideline-based indications to treat. Hemoglobin trajectory and a history of heart failure appear to guide treatment start. These results support the concept that anemia is sub-optimally managed among NDD-CKD patients in the real-world setting.

## Introduction

Anemia is a frequent complication in non-dialysis-dependent chronic kidney disease (NDD-CKD) patients and is associated with decreased quality of life, as well as with increased morbidity and mortality^1,2^. The pathogenesis of anemia in CKD is multifactorial and complex, but iron deficiency (ID) and a relative decrease in erythropoietin production are the most important mechanisms involved. These pathophysiological processes are the basis for the current management of CKD anemia, where erythropoiesis stimulating agents (ESAs) and iron supplementation represent the cornerstone of NDD-CKD anemia treatment^3,4^. Though replacement therapy with ESAs has been available for over 30 years, the optimal algorithm for anemia management, including triggers of treatment initiation, medication choice, and target biochemical levels to avoid possible adverse effects, remains controversial, particularly in the pre-dialysis phase of CKD^5–8^. Further controversy in defining ideal management of anemia in NDD-CKD was introduced by the results from clinical trials, which indicated higher cardiovascular mortality associated with hemoglobin levels higher than 11 g/dL when in use with ESAs. These findings led to a black box warning issued in 2007 by the United States Food and Drug Administration^9^.

In general terms, the triggers of anemia treatment initiation are, currently, low hemoglobin levels (for ESA use) and biochemical evidence of ID (for iron replacement)^10^. Current guidelines leave room for practice variation in treatment initiation, choice of medication, and laboratory targets of therapy^11^. Not surprisingly, existing literature indicates that practice patterns in the real-world setting vary substantially from current recommendations, for reasons yet to be investigated. A substantial proportion of NDD-CKD patients with low hemoglobin levels and/or with ID are not treated^11,12^. In a previous cross-sectional analysis of the Chronic Kidney Disease Outcomes and Practice Patterns Study (CKDopps)^11^, the prevalence of ESA prescriptions for individuals with hemoglobin < 10 g/dL was 28% in the USA, 39% in Brazil, and 57% in Germany; among patients with ferritin < 100 ng/mL or TSAT < 20% who also had hemoglobin < 12 g/dL, 27–44% of patients were prescribed iron supplementation. Variation in anemia management across regions and patient groups may relate to several reasons, including regional differences in practice recommendations, regulatory guidance, safety concerns, access to and cost of medications, logistical challenges of administering parenteral therapies, and differences in patient comorbidity profile^10,13,14^.

A comprehensive longitudinal description of variations in multinational clinical practice patterns of anemia management, including new prescriptions of ESAs and iron replacement, for NDD-CKD patients is lacking in the literature. The objective of this analysis is to describe the factors associated with anemia treatment initiation, and hemoglobin trajectories during longitudinal follow-up in the real-world setting among patients with NDD-CKD Stage 3 to 5 under nephrology care.

## Materials and methods

### Study sample

CKDopps is an ongoing international prospective cohort study of adult NDD-CKD patients with estimated glomerular filtration rate (eGFR) < 60 ml/min/1.73m^2^ under nephrology care in Brazil, France, Germany, Japan, and the US. The study design and protocol details have been published previously^15^. Participants were sequentially or randomly selected from stratified national samples of nephrologist-run CKD clinics. Routine laboratory and medication-related data were collected longitudinally throughout the study, up to a monthly frequency. Clinical data were transcribed from medical records in Brazil and the US, and via abstraction from the electronic health records in Germany. CKDopps was approved by an independent institutional review board (*E&I Review Services)*, along with national and/or local ethics committees, as required by local ethics regulations. The study was conducted with adherence to the Declaration of Helsinki. Written, informed consent was obtained from all patients eligible for study participation. Protocols, data storage, and data use were compliant with international data privacy laws.

The current analysis used data from phase 1 of CKDopps (2013–2018) in Brazil, Germany, and the US, along with CKDopps phase 2 data (2018–2019) only for the US. Japan data were not included in this analysis, since data collection started later in Japan and data are still not available. Since the study protocol in France differs in terms of frequency of study visits, data were not included for consistency. We included patients who were not using any anemia treatment (oral iron, intravenous [IV] iron, or ESAs) within 6 months prior to enrollment. Patients were excluded if baseline demographics or the medical history questionnaire were lacking, or if no laboratory and medication data were reported. We utilized longitudinal data to classify patients in the analysis sample as “new users” or “never users,” based on whether they received a new prescription for oral iron, IV iron, or an ESA during study follow-up. We defined anemia as Hb < 12 g/dL, and used Hb < 10 g/dL and < 9 g/dL as indicators of low hemoglobin. ID was considered if patients had TSAT < 20% or ferritin < 100 ng/mL.

We carried forward the recorded treatment status until the next available medication information for patients whom a clear indication of treatment discontinuation was not identified in the database. Moreover, we took into consideration in the analysis the expected duration of treatment for each therapy (e.g., up to a maximum of 3 months for ESA and IV iron use and 12 months for oral iron use). The most recent laboratory values within 12 months prior to treatment initiation were selected for the analysis.

In addition, we describe clinic-level targets for Hb and their associations with achieved Hb levels in patients with advanced CKD receiving anemia treatment. Clinic-level Hb upper and lower targets were collected from nephrologist surveys and averaged if multiple nephrologists in a clinic responded to the survey.

### Statistical analysis

Baseline demographic and clinical characteristics of patients were summarized by country and according to use/non-use of anemia treatment after study enrollment. Sub-distribution hazards and risk expressed by cumulative incidence function (CIF) of treatment initiation were estimated separately (univariately) for each type of treatment, by hemoglobin, TSAT, ferritin, and CKD stage at baseline. Time at risk extended from study enrollment until the initiation of treatment, and was censored at the earliest of patient switch to a different level of the stratification variable (i.e., CKD stage, hemoglobin/TSAT/ ferritin), date of last available medication data, or end of patient follow-up. The CIF model treated death and end-stage renal disease (i.e., dialysis and transplantation) as competing events and estimated the probability that treatment initiation would happen prior to the competing events. Similarly, CIFs were also estimated for each type of treatment by comorbidity/demographic patient subgroup status at baseline. We included as stratification factors the presence of any preexisting cardiovascular disease (coronary artery disease [CAD], heart failure [HF], and diabetes mellitus [DM]), age (< 70 vs. ≥ 70), sex, and race (black vs. non-black). We used the Fine and Gray method^16^ to test for differences in CIF between groups and the Benjamini–Hochberg method to control for false discovery rate and multiple comparisons of *p* values^17^.

A linear mixed regression model with random intercepts and random slopes was used to estimate individual hemoglobin slopes, from study enrollment until the end of follow-up for never users whereas a piecewise linear mixed model with discontinuity at time of treatment was used to estimate pre-treatment and post-treatment hemoglobin slopes for new users. This modeling approach accounts for the correlation between repeated hemoglobin measurements within patients during follow-up. We observed an approximately normal distribution of slopes from individual linear regressions, meeting a key assumption of random effects in the regression method. The distributions of individual hemoglobin slopes were presented as box-plots, by CKD stage and treatment status. Average hemoglobin trajectories were plotted using penalized b-splines for the same subject groups.

Among new users of anemia treatment, we additionally summarized biochemical parameters captured before initiation of anemia treatment and Sankey diagrams were used to illustrate treatment switch and discontinuation patterns at 3, 6, 9, and 12 months after prescription. For the analysis used to create the Sankey plot, monthly data were aggregated to the 3-month level such that treatment during any month of the interval was considered active for the interval.

All statistical analyses were conducted using SAS, version 9.4 (SAS Institute Inc., Cary, NC).

## Results

**Figure 1:**
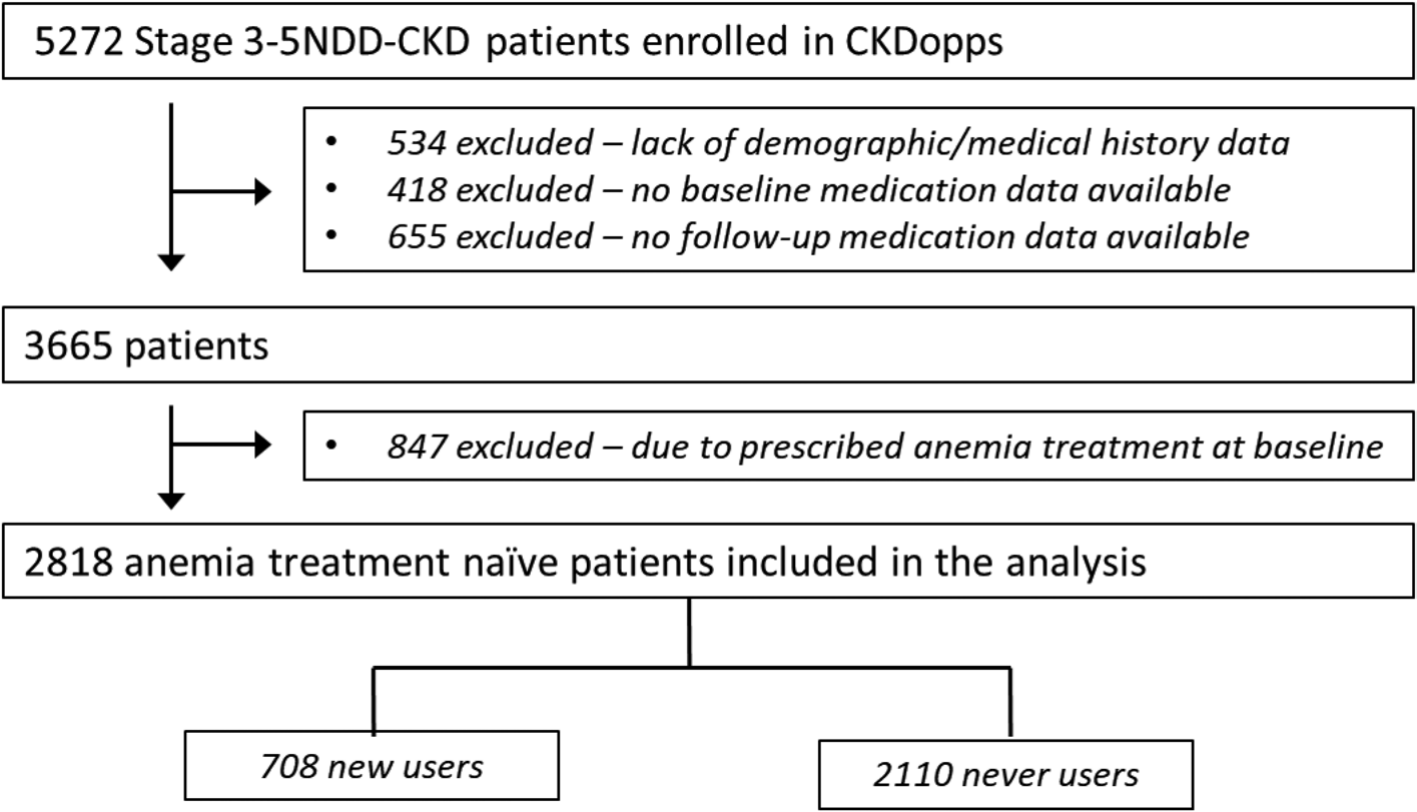
Consort diagram of the CKDOPPS cohort with patients from Brazil, Germany, and the United States.

The source population consisted of 5272 patients with NDD-CKD Stage 3 to 5 with follow-up in nephrologist-run clinics in Brazil (18%), Germany (48%), and the US (34%). We excluded 534 patients who lacked demographic or medical history data, 418 patients without baseline medication data, 655 without follow-up medication data, and 847 patients who were receiving any anemia treatment at study enrollment (see CONSORT diagram, Fig. 1). A total of 2818 patients who were using any anemia treatment medication for 6 months were selected for our final sample. Median follow-up time was 25.8 (14.6, 40.8) months; follow-up differed by country (median years was 1.9 years in Brazil, 3.2 years in Germany, and 1.2 in the US).

During follow-up, 708 patients (25%) became new users of anemia treatment; the remaining 2110 (75%) were never users. Among the new users, 52% were prescribed oral iron, 18% IV iron, and 38% ESAs for their initial anemia treatment (either alone or in combination). The baseline characteristics of new users and never users are provided in Table 1. New users had lower eGFR (25.6 ml/min/1.73 m^2^ [9.1] vs. 28.7 ml/min/1.73 m^2^ [10.6]), Hb (11.7 g/dL [1.5] vs. 12.8 g/dL [1.8]), TSAT (22.2%[9.5] vs. 24.4% [9.4]) and ferritin (120 ng/dL [61,250] vs. 129 ng/dL [62,246]) levels than never users.

**Table 1:** Baseline patient characteristics of anemia treatment, by new user or never user status after study enrollment.

	Overall		Brazil		Germany		US	
	New users	Never users	New users	Never users	New users	Never users	New users	Never users
Patients, N	708	2110	90	348	417	717	201	1045
**Demographics**								
Age, years	69.7 (12.9)	69.0 (13.0)	66.9 (15.8)	65.2 (13.5)	71.1 (11.9)	72.0 (12.1)	68.1 (13.0)	68.2 (12.9)
Female sex, %	45%	43%	53%	42%	38%	40%	56%	45%
Black race, %	11%	13%	30%	24%	–	–	25%	18%
Body mass index, kg/m^2^	29.7 (6.1)	30.7 (6.5)	28.0 (4.8)	28.6 (5.5)	29.0 (5.5)	29.7 (5.4)	32.1 (7.2)	32.1 (7.2)
CKD vintage	4.8 (5.1)	4.9 (4.9)	4.7 (5.5)	4.5 (4.8)	–	–	4.9 (5.0)	5.0 (4.9)
**Comorbidities, %**								
Coronary heart disease	28%	27%	26%	20%	29%	27%	28%	29%
Heart failure	16%	13%	21%	10%	13%	13%	19%	14%
Other cardiovascular disease	22%	19%	20%	13%	23%	20%	22%	21%
Diabetes	50%	49%	47%	47%	46%	42%	58%	53%
Cirrhosis	2%	2%	0%	1%	3%	2%	2%	1%
GI Bleed	3%	1%	3%	2%	3%	1%	2%	1%
Cancer	15%	15%	12%	10%	14%	14%	17%	16%
Laboratory*								
eGFR, ml/min/1.73 m^2^	25.6 (9.1)	28.6 (10.5)	23.3 (9.2)	29.0 (10.9)	26.4 (8.6)	29.9 (10.7)	24.9 (9.9)	27.6 (10.2)
Hemoglobin, g/dL	11.7 (1.5)	12.8 (1.8)	11.6 (1.6)	13.0 (1.8)	11.9 (1.4)	13.1 (1.5)	11.1 (1.6)	12.4 (1.8)
Ferritin, ng/mL	119 [61,250]	128 [61,239]	121 [51,328]	165 [84,301]	120 [65,242]	122 [65,230]	111 [47,250]	122 [53,243]
TSAT, %	22.2 (9.5)	24.3 (9.5)	27.5 (11.5)	27.5 (9.8)	21.8 (9.1)	24.3 (9.5)	20.6 (8.9)	22.1 (8.7)

*Most recent value within 6 months prior to the study enrollment.

Results presented as Number, mean (SD), median [IQR] or percentage as appropriate.

Among new users, laboratory and treatment data at the time of treatment initiation are reported in Supplemental Table 1. Patients starting oral iron had higher mean eGFR than those starting IV iron or ESA users (24.7 ml/min/1.73 m^2^ for oral iron, 20.6 ml/min/1.73 m^2^ for IV iron, 18.5 ml/min/1.73 m^2^ for ESA). New ESA prescriptions (alone or in combination with iron replacement) occurred at lower hemoglobin (10.2 g/dL vs. 11.2 g/dL for oral iron and 10.9 g/dL for IV iron), at higher ferritin (180 ng/mL vs. 90 ng/mL for oral iron and 101 for IV iron), and at higher TSAT levels (23.2% vs. 19.1% for oral iron and 19.3% for IV iron) compared to new use of iron replacement. A lower proportion of patients had TSAT < 20% for ESA users (37%) compared to those on use of oral (62%) and IV iron (64%). The proportion of oral iron users who were at CKD Stage 3 (25%) was much higher than those on parenteral therapies: 13% of IV iron users were at Stage 3, as well as 9% of ESA users. CKD Stage 4 was when most of the patients started therapy: 59% of oral iron and IV iron users and 50% of ESA users. In CKD Stage 5, the proportion of patients who were started on oral iron was much lower (16%) than IV iron (29%) and ESA users (41%). Patient characteristics at treatment initiation by medication and country can be seen in Table 2.

**Table 2:** Laboratory values and treatment data at treatment initiation among new users, by country and type of treatment started.

	Brazil			Germany			US		
	Oral Iron	IV Iron	ESA	Oral Iron	IV Iron	ESA	Oral Iron	IV Iron	ESA
Patients, N	53	18	32	181	93	178	136	27	48
% on monotherapy	94%	44%	59%	92%	73%	83%	95%	81%	83%
Laboratory values*									
CKD stage									
Stage 3	23%	11%	19%	22%	13%	6%	30%	14%	13%
Stage 4	66%	39%	42%	62%	61%	54%	52%	64%	41%
Stage 5	11%	50%	39%	16%	26%	39%	18%	23%	46%
eGFR, ml/min/1.73m^2^	25.0 (9.9)	18.2 (11.8)	19.8 (10.3)	24.2 (10.2)	21.2 (9.2)	18.1 (7.5)	25.3 (11.5)	19.8 (8.1)	18.8 (9.2)
Hemoglobin, g/dL	11.5 (1.3)	10.4 (1.6)	10.9 (1.6)	11.2 (1.5)	11.2 (1.4)	10.2 (1.3)	11.1 (1.6)	10.2 (1.7)	9.6 (1.4)
Ferritin, ng/mL	99 [50,281]	100 [90,162]	143 [98,346]	89 [46,171]	110 [51,191]	180 [90,362]	87 [50,247]	61 [30,225]	200 [133,370]
Ferritin < 100 ng/mL	52%	43%	25%	58%	48%	28%	55%	58%	17%
TSAT, %	28.5 (11.5)	19.3 (9.7)	28.3 (10.5)	17.8 (9.0)	20.0 (11.1)	21.8 (8.7)	18.4 (9.1)	15.6 (7.0)	29.4 (10.1)
TSAT < 20%	18%	60%	17%	69%	61%	43%	60%	82%	13%
**ESA type**									
Patients with ESA type data, N	2	10	29	10	21	178	5	3	40
Darbepoetin alfa	0%	0%	3%	30%	62%	54%	40%	0%	65%
Epoetin alfa	100%	100%	97%	60%	24%	25%	60%	67%	33%
Epoetin beta – pegylated	0%	0%	0%	10%	19%	22%	0%	33%	3%

Patients who are under combined treatment regimen could contribute to any of three columns.

*The most recent value within 12 months prior to the treatment initiation.

Results presented as Number, mean (SD), median [IQR] or percentage as appropriate.

**Figure 2:**
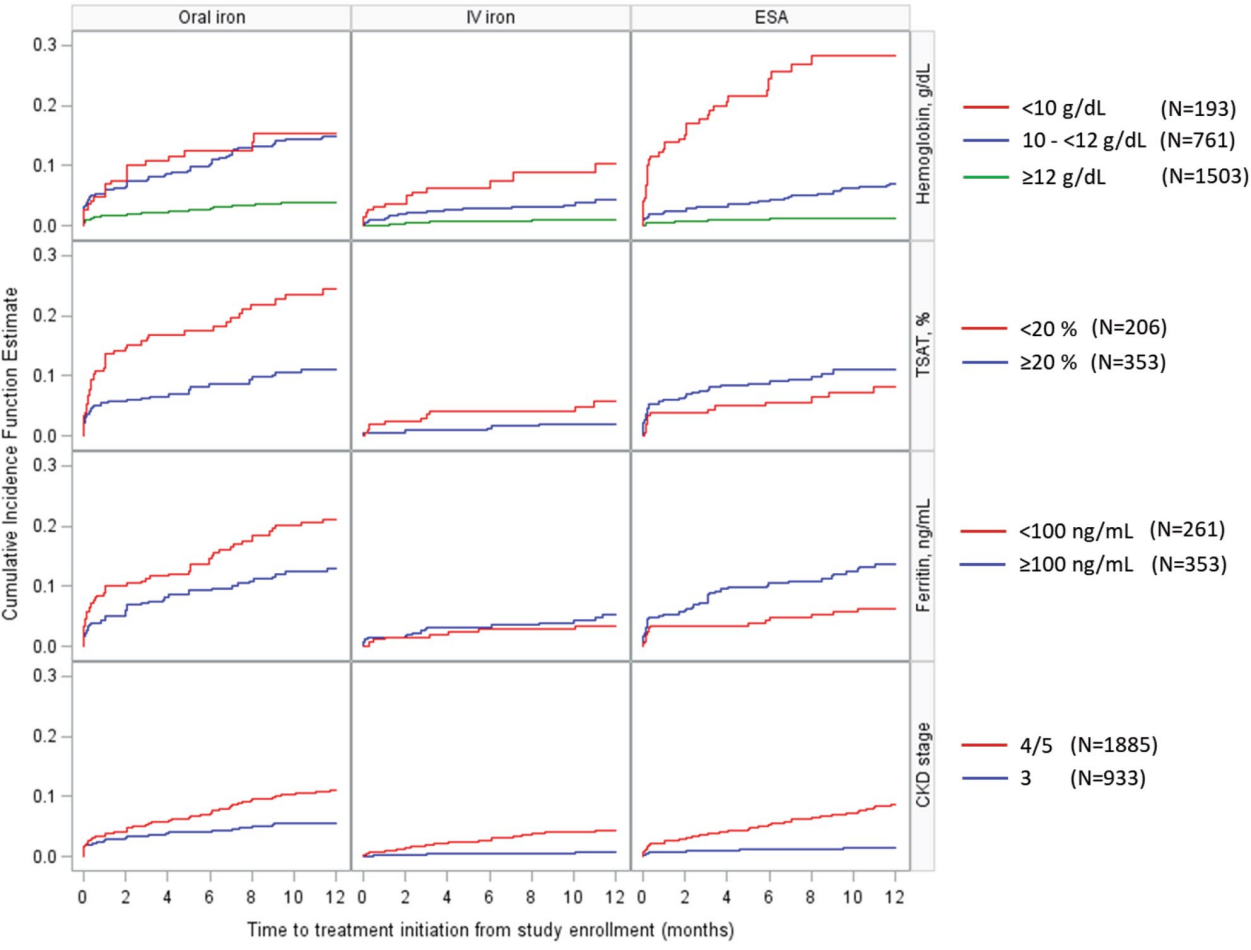
Cumulative incidence function (CIF; 0–1) of anemia treatment initiation from study enrollment, stratified by baseline anemia labs or CKD stage.

The CIF of any treatment (oral or IV iron, ESA) initiation in the 12 months (Table 3) after enrollment was 18% overall at 12 months, and was 40% for patients with Hb < 10 g/dL, 24% for patients with Hb 10 to < 12 g/dL, and 6% for Hb ≥ 12 g/dL. The 12-month CIF of any treatment by CKD stage was 19% for Stage 4 or 5, and 7% for Stage 3. One-third of patients with Hb < 9 g/dL (33%) and 28% of individuals with Hb < 10 g/dL had an ESA start during the 12 months after enrollment. One-quarter of patients with TSAT < 20% were treated with oral iron (CIF at 12 months after enrollment: 26%), and a much smaller proportion were prescribed IV iron (CIF: 6% for TSAT < 20% and 4% for ferritin < 100 ng/mL). Higher iron treatment start CIFs were seen for patients with low Hb levels for oral iron (CIF at 12 months: 24% for Hb < 10 g/dL vs. 18% for Hb 10 to < 12 g/dL and 5% for Hb ≥ 12 g/dL) and IV iron (12% for Hb < 10 vs. 6% for Hb 10 to < 12 g/dL and 2% for Hb ≥ 12 g/dL). In patients with Hb < 10 g/dL, ESAs were more likely prescribed to patients with higher iron parameter levels; the CIF at 12 months was 53% for patients with TSAT ≥ 20% and 22% for patients with TSAT < 20%. The CIF curves of anemia treatment initiation by hematimetric indices are shown in Fig. 2.

**Table 3:** Cumulative incidence (95% CI) of new anemia treatment at 12 month follow-up.

	N	Any treatment	Oral iron	IV iron	ESA
***Demographics***					
**Age at baseline**					
< 70	1252	14% (12%, 16%)	8% (7%, 10%)	3% (2%, 4%)	6% (4%, 7%)
≥ 70	1566	17% (15%, 19%)	10% (8%, 11%)	4% (3%, 5%)	7% (6%, 8%)
**Gender**					
Female	1222	17% (15%, 20%)	**11% (9%, 13%)**	4% (3%, 5%)	6% (5%, 8%)
Male	1596	14% (13%, 16%)	**8% (7%, 9%)**	3% (2%, 4%)	6% (5%, 8%)
**Race**					
Black	352	19% (15%, 24%)	**14% (11%, 18%)**	3% (2%, 6%)	6% (3%, 9%)
Non-black	2456	15% (14%, 17%)	**8% (7%, 10%)**	3% (3%, 4%)	6% (5%, 7%)
***Comorbidities at baseline***					
**Cardiovascular disease**					
No	1660	15% (13%, 16%)	9% (7%, 10%)	3% (2%, 4%)	6% (5%, 7%)
Yes	1153	17% (15%, 20%)	10% (9%, 12%)	4% (3%, 5%)	7% (5%, 8%)
**Coronary Artery Disease**					
No	2055	15% (14%, 17%)	9% (8%, 10%)	3% (2%, 4%)	6% (5%, 7%)
Yes	756	17% (14%, 20%)	10% (8%, 12%)	5% (4%, 7%)	6% (5%, 8%)
**Heart failure**					
No	2427	**14% (13%, 16%)**	**9% (7%, 10%)**	3% (3%, 4%)	**6% (5%, 7%)**
Yes	380	**23% (19%, 28%)**	**14% (11%, 18%)**	5% (3%, 7%)	**9% (6%, 12%)**
**Diabetes**					
No	1442	15% (13%, 16%)	9% (7%, 10%)	3% (2%, 4%)	5% (4%, 7%)
Yes	1372	17% (15%, 19%)	10% (8%, 12%)	4% (3%, 5%)	7% (6%, 9%)
***Baseline anemia laboratories and CKD stage***					
**Hemoglobin, g/dL**					
< 10	193	**40% (32%, 48%)**	**15% (10%, 22%)**	**10% (5%, 17%)**	**28% (21%, 36%)**
10—< 12	761	**24% (20%, 27%)**	**15% (12%, 18%)**	**4% (3%, 6%)**	**7% (5%, 9%)**
≥ 12	1503	**6% (5%, 7%)**	**4% (3%, 5%)**	**1% (1%, 2%)**	**1% (1%, 2%)**
**TSAT, %**					
< 20	206	30% (24%, 37%)	**24% (18%, 31%)**	6% (3%, 10%)	8% (4%, 13%)
≥ 20	353	21% (17%, 26%)	**11% (8%, 15%)**	2% (1%, 4%)	11% (8%, 15%)
**Ferritin, ng/mL**					
< 100	261	26% (21%, 32%)	21% (16%, 27%)	3% (2%, 6%)	6% (4%, 10%)
≥ 100	353	26% (22%, 31%)	13% (9%, 17%)	5% (3%, 8%)	14% (10%, 18%)
**CKD stage**					
Stage 3	933	**7% (6%, 9%)**	**6% (4%, 7%)**	**1% (0%, 2%)**	**1% (1%, 2%)**
Stage 4/5	1885	**19% (17%, 21%)**	**11% (10%, 13%)**	**4% (3%, 5%)**	**8% (7%, 10%)**

Bold numbers represents *p* value < 0.05 for Benjamini–Hochberg adjustment for differences.

*CIF* cumulative incidence function, *TSAT* transferrin saturation, *CKD* chronic kidney disease.

**Figure 3:**
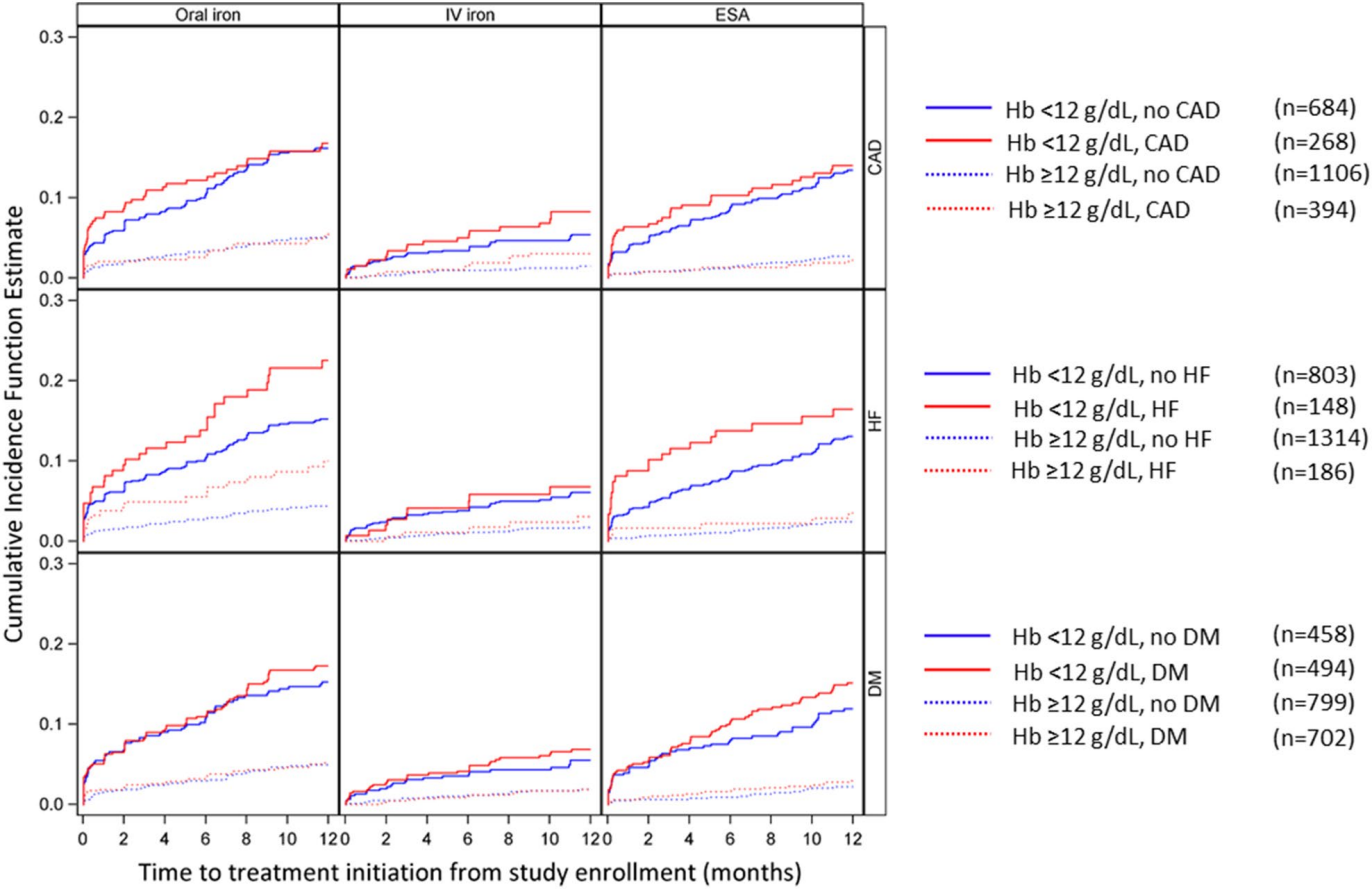
Cumulative incidence function (CIF; 0–1) of anemia treatment initiation from study enrollment, stratified by Hb level and comorbidity status.

Stratification (Fig. 3) of the 12-month CIF curves by comorbidities and demographics showed that a small proportion of patients with HF received IV iron therapy (CI 5% [3%, 8%]), not restricted to the iron deficient HF patients. Oral iron was prescribed more frequently than IV iron, with a higher CIF for persons with versus without HF (CIF 14% vs. 9%, p-value of difference < 0.01). Among patients with CAD, the CIF was low for oral iron (10%), IV iron (5%), and ESA use (6%), and CIFs were similar for persons with versus without CAD (*p* > 0.2 for all treatment types). The 12-month CIF results stratified by other clinical and demographic variables are in Table 3.

**Figure 4:**
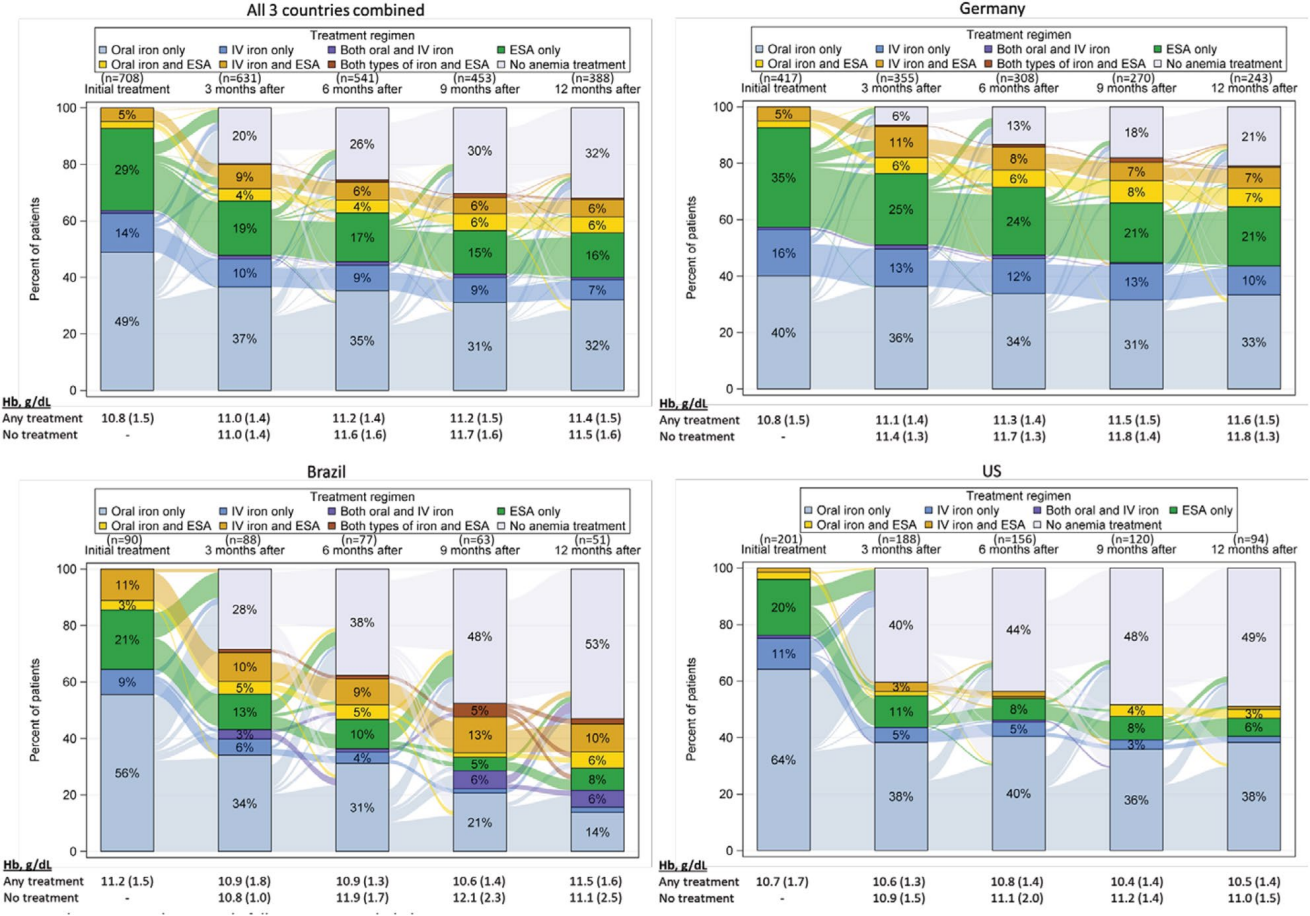
Sankey plot of treatment patterns at 0, 3, 6, 9, and 12 months after treatment initiation for ‘new users during study follow-up, by country.

Longitudinal treatment patterns by country are provided in Fig. 4. Approximately half of new users were no longer on any anemia therapy one year later in Brazil (54%) and the US (48%), compared to 21% in Germany. Oral iron alone was the most common first treatment option in the US (64%); in Germany ESA (35%) and IV iron (16%) prescriptions were notably higher than in the US and Brazil. Combination therapy was uncommon during follow-up, and more common at the time of new use in Brazil (11% for the combination of IV iron and ESA and 3% for oral iron and ESA). Hemoglobin levels were similar in those who remained on treatment a year after initiation (mean Hb at 12 months was 11.6 g/dL for treated patients in Germany, 11.1 in Brazil, and 10.6 in the US). Patients who were dropped from treatment had higher Hb levels than patients who remained on treatment in all three-month intervals of the 12-month period, in Brazil (11.6 g/dL for patients no longer being treated vs. 11.1 g/dL for patients with treatment at month 12), the US (10.9 g/dL vs. 10.6 g/dL), and Germany (11.8 g/dL vs. 11.6 g/dL).

**Figure 5:**
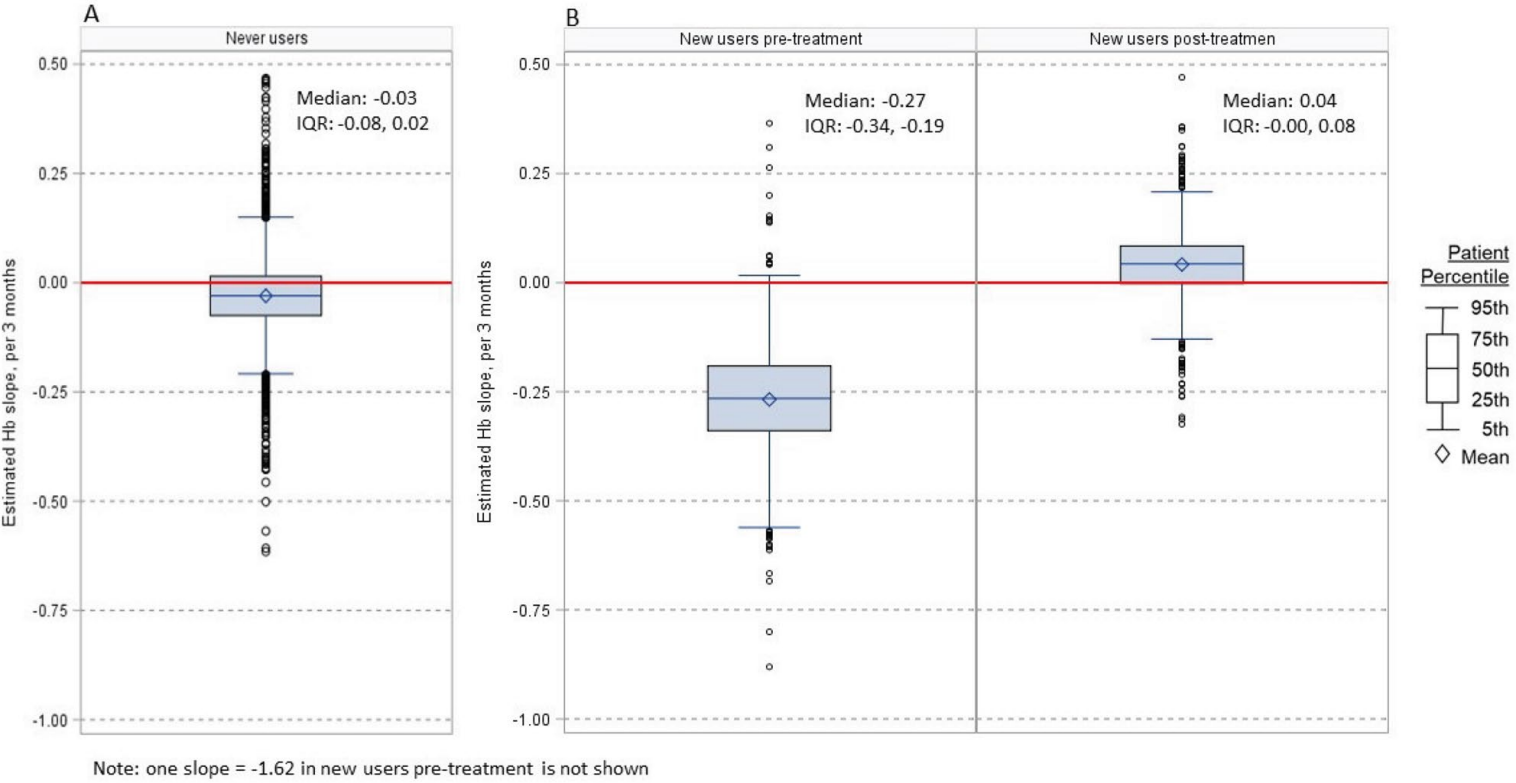
Distribution of within-person individual hemoglobin trajectories, during follow-up, expressed as slope per 3 months for ‘never users’ (**A**), new users pre and post anemia treatment (**B**).

Figure 5 shows the distribution of hemoglobin trajectory slopes. The majority of patients who never used anemia treatment had stable hemoglobin levels (Fig. 5A), with a median [IQR] hemoglobin slope of − 0.04 [0.2, 0.09] g/dL per 3 months of follow up. New users had a larger drop in hemoglobin levels before becoming new users in our analysis, with a median [IQR] Hb slope − 0.26 [− 0.5, − 0.11] per 3 months (Fig. 5B, left panel). After starting treatment (Fig. 5B, right panel), most patients had modestly positive slopes, but 25% had a negative Hb trajectory (median [IQR]: 0.02 [− 0.19, 0.21] per 3 months). Additional hemoglobin trajectories along with stratified cumulative incidence analyses can be found in the supplemental material.

The results from the survey for hb targets, applied to 44 clinics: Brazil (n = 12 clinics), Germany (n = 15 clinics), US (n = 17 clinics). Patients from clinics reporting higher Hb thresholds presented with higher Hb levels across countries. On average, the facilities with Hb ≥ 10 g/dL, as the target for lower levels of Hb during anemia treatment, produced hb levels of 10.7, 11, and 11.5 g/dL in the US, Brazil, and Germany, respectively.

## Discussion

In this real-world study of patients with moderate to severe NDD-CKD under nephrology care, we identified significant undertreatment of anemia, especially in patients with Hb < 10 g/dL. The results of this longitudinal analysis confirm and extend findings presented in our previous cross-sectional analysis^11^. We found that the majority of NDD-CKD patients who are followed by nephrologists and have low hematinic measures are not given conventional anemia medication within a year. We also report a high proportion of discontinuation (either permanent or intermittent) within one year of initial anemia prescription, as well as noticeable practice variation across countries.

The current standard of CKD anemia therapy relies on prescription of iron supplementation, ESAs, and, rarely, red blood cell (RBC) transfusions as treatment options. The initiation of therapy is determined by either severity of anemia (i.e., low Hb levels) or ID^18–20^. In this multinational study based in nephrology clinics, we observed that oral iron therapy is the medication most often prescribed, either alone or in combination with an ESA and that treatment initiation is guided mainly by hemoglobin levels and, to a lesser extent, by iron stores. The cumulative incidence curves further illustrate that earlier starts are more likely at lower Hb and eGFR levels, as well as in the presence of comorbidities. Patients with lower eGFR customarily have more frequent clinic visits and are assessed with more laboratory values, as indicated by Wong et al.^11^, and thus the initiation of anemia therapy becomes more likely. These findings may help to improve anemia management for NDD-CKD patients.

This study also illustrates the underutilization of iron replacement therapies. The preferred route of iron replacement was oral, in this population with a high incidence of ID; from a patient prognosis perspective, this choice of prescription differs with the findings of a randomized clinical trial that reported superiority for IV iron therapy to oral iron in the correction of ID^21^. Guidelines often recommend a trial of iron for anemic patients with TSAT < 30% to avoid iron-restricted erythropoiesis^10^; our data show that this population is not commonly treated. Yet more strikingly, most patients with overt laboratory evidence of ID were not treated during the observation period. We noted that IV iron is usually prescribed at more advanced stages of CKD, due to the decrease in iron absorption via the GI tract and higher frequency of GI side effects with oral iron use as CKD progresses to the advanced stages^22^. The progressive increases in inflammation, as well as hepcidin, with CKD progression also results in the lack of efficacy of oral iron use. IV iron prevents the occurrence of iron-restricted erythropoiesis more effectively than oral iron^19,23^; the lack of IV iron use in NDD-CKD patients may lead to lower hemoglobin levels and higher ESA and IV iron doses during end-stage renal disease^24,25^. In the FIND CKD trial, IV ferric calboxymaltose targeting higher iron parameter levels (achieved ferritin = 503 ng/mL; TSAT = 31.2%) resulted in greater Hb levels, reducing or delaying the need for anemia medications (ESAs or blood transfusions) other than oral iron^21^. In addition, a DOPPS study of incident HD patients, evaluating use of ESAs, demonstrated a positive association of initial ESA and IV iron doses with mortality^26^. Compared to patients with Hb < 10 g/dL and ESA dose between 5000 and 10,000 units per week, the HR for all-cause mortality for those prescribed ESA dose > 25,000 units per week was 1.54 (1.01–2.36); these data support the possibility that proactively treating anemia prior to dialysis start may improve outcomes after dialysis initiation^26^. We expect that the improvement in iron replacement for NDD-CKD patients will reduce the high dose of ESA and iron medication seen at the start of renal replacement therapy. Our current analysis illustrates the unmet need to improve adherence to anemia practice guidelines in NDD-CKD, and this may in turn limit doses of ESA and iron given at the start of renal replacement therapy. Doing so may plausibly help to lower the high mortality seen in the early dialysis period, though additional studies evaluating this possibility are needed.

Similar to findings in our previous cross-sectional analysis, ESA prescription was uncommon, even for patients who are at hemoglobin levels associated with adverse events^11^. Most guidelines emphasize that ESAs should be started and maintained at the lowest possible dose in order to support hemoglobin levels while avoiding the risks associated with RBC transfusions or a need for high ESA doses in the future^10^. Gilbertson et al. reported that the risk of RBC transfusions increases in HD patients when the level of hemoglobin is < 10 g/dL^27^; in our study, patients who were below this threshold had fewer ESA starts, with a CIF at 12 months of only 28% (21%, 36%). Lawler et al. demonstrated a similar Hb threshold for NDD-CKD patients Stage 3 to 5 in a study of 97,636 patients in the Veterans Administration Healthcare System; this analysis also showed that the use of ESAs and/or iron, at any hemoglobin level, was responsible for a considerable attenuation of the risk for red blood cell transfusions^28^. Practice patterns of anemia treatment from facilities that target higher Hb levels are directly associated with achieved Hb, but the impact of this practice on complications (i.e., transfusions) or other adverse outcomes needs to be investigated. Higher hemoglobin levels, as a result of ESA treatment, could lead to adverse effects^6,29–32^, and current guidelines have clearly recommended delineated ranges of Hb, where treatment provides benefit. The potential risk of overcorrecting Hb, thus exposing patients to adverse effects, may account not only for the late and infrequent ESA starts, but also for the abounding number of discontinuations during follow-up^8,18^. On the other hand, it is plausible that treatment discontinuation may reflect a reaction to achievement of treatment goals for both iron replacement and ESAs protocols, particularly favored by the intermittent nature of CKD care in the non-dialysis setting. To avoid potential negative hemoglobin trajectories, a strategy that allows longer treatments at lower doses should be tested in future studies.

Our stratified analyses indicated differences in treatment initiation by patient characteristics, such as male sex, black race, and coronary artery disease (particularly HF), but overwhelmingly indicated undertreatment even in the presence of comorbidity-specific indications. Patients with HF had higher one year probability of oral iron prescription (14% vs. 0.9%), where there’s noticeable separation of ESA starts for the anemic HF patients (overall 9% vs. 6%). It’s worth calling attention to the HF literature, which indicates that oral iron treatment may not improve outcomes. High-dose oral iron did not improve exercise capacity over 16 weeks in patients with reduced left ventricular ejection fraction (< 40%) and ID in the IRON OUT HF trial^33^. Due to the low frequency of IV iron use, we were unable to detect differences in treatment starts, based on the HF status. The low number of HF patients treated for anemia and ID in our results highlights a potential area for improvement, as intravenous iron has been shown to improve cardiac function, functional capacity, symptoms, hospitalizations, and quality of life independently of their hemoglobin levels^19,34–38^. Earlier initiation of CKD anemia treatment in patients with a preexisting diagnosis of heart disease would typify treatment by indication^37–39^; however, it is possible that anemia and ID treatment may be triggered by a higher burden of symptoms observed in patients with HF.

We also observed interesting findings related to the trajectories of hemoglobin in our longitudinal analysis. The distribution of Hb slopes were distinct in patients who were not treated throughout the entire follow-up period. Most patients who never received anemia medication, during follow-up, maintained flat hemoglobin slopes. For those who started treatment, Hb slopes differed before and after treatment initiation: they trended downwards during the months preceding anemia treatment initiation, but after treatment, for the majority of patients Hb levels rose to the recommended target range. Aligned with current recommendations from the latest KDIGO guidelines, negative slope in Hb appeared to be associated with anemia treatment initiation, suggesting that a longitudinal decrease in Hb levels may be taken into account by clinicians when defining initiation of therapy^10^. After treatment, flat or negative Hb slopes, denoting a lack of response to treatment, were much less frequent. Additionally, most patients who started anemia treatment increased hemoglobin levels after treatment initiation, indicating that, in real-world practice, the therapeutic options are effective in correcting anemia in patients with moderate to advanced CKD. In Germany, where patients were started more often on parenteral therapies, the mean hemoglobin after a year of follow-up was at least one point higher than the US, where ESAs and IV iron were used less often.

CKDopps is well suited to capture practice patterns amongst countries, and there were many interesting country differences that provide insights regarding variation in anemia management practices. In Germany, where the use of ESAs and IV iron was more common, hemoglobin and iron store levels for patients who remained on treatment were the highest among the countries in the study; this association suggests that higher use of IV iron and/or less restricted ESA use may more efficiently achieve laboratory goals for anemia management, while also avoiding potential adverse effects**.** Patients in the US and Brazil had far more oral iron prescriptions and, at the end of follow-up, the majority of patients were no longer on any anemia medication for reasons yet to be clarified. ESA and iron use in patients with Hgb < 11 g/dL were more common in Brazil and Germany, and the mean Hb at ESA start was higher in Brazil (10.9 g/dL) and Germany (10.2) than in the US (9.6), possibly due to differences in regional guidelines, as well as different norms for medication access in those countries^40,41^, as well as physician-led decisions and practice preferences. A previous analysis from the CKDopps nephrologist practice survey indicates that the most common hemoglobin threshold for starting ESA therapy significantly varies across countries, particularly in countries with a more emphatic restriction of ESA therapy^42^. To the best of our knowledge, this is the first analysis that explores real-world country differences in longitudinal anemia management in NDD-CKD patients.

Readers should interpret these results in the context of the study design. Although the observational design does not allow us to make causal inferences, it is appropriate to address the stated goals to describe patterns and triggers of CKD anemia treatment in current practice. The standardized data collection protocol and stratified random sampling method used to recruit facilities allows us to make inferences about practice patterns that may generalize broadly to the countries represented. Patients’ heterogeneous frequencies of clinic visits and laboratory measures likely drove differences in anemia treatment by CKD stage, but this became more common with decline in kidney function, and it is likely that differences in frequency of follow-up influenced treatment choices. This reflects the reality of real-world practice, where most patients had either few or sparse visits which could have resulted in missing values for labs and treatment. There were also notable country differences in timing of medication data collection that were accounted for in the analysis: since Germany aggregated their medication data by calendar quarter we did the same for the US and Brazil, which had been collected monthly. To our knowledge, this is the first study that uses real-world longitudinal data to evaluate the impact of patient characteristics in anemia management, while also tracking the response in hemoglobin and iron stores of NDD-CKD patients. Studies that focus on the impact of patient characteristics and their symptom burden associated with anemia, as possible triggers for treatment initiation, are needed.

## Conclusion

In summary, this multinational analysis highlights the low proportion of treatment initiation for anemia with existing conventional therapies for advanced NDD-CKD patients, even at levels of Hb and iron parameters that are indications for anemia treatment according to clinical guidelines, and among patients with comorbidity-specific indications for treatment. Many patients discontinue anemia treatment, with these agents, within months after starting, for reasons yet to be clarified. A downward hemoglobin trajectory and a history of heart failure appear to trigger anemia treatment. These results add to the body of evidence that anemia is sub-optimally managed among patients with NDD-CKD, especially in those with an Hb < 10. The introduction of novel anemia treatment strategies and therapies (i.e., HIF stabilizers) that offer better efficacy of reaching targets while offering less adverse effects to patients is to be determined in future studies.

## Supplementary Information

Supplementary Information.

## Data Availability

The datasets generated during and/or analysed during the current study are available from the corresponding author on reasonable request.
