# Supplementary material for: A real-world longitudinal study of anemia management in non-dialysis-dependent chronic kidney disease patients: a multinational analysis of CKDopps
Source: Sci Rep. 2021 Jan 19;11:1784. doi: 10.1038/s41598-020-79254-6 (PMC7815803; doi:10.1038/s41598-020-79254-6)
Supplement: Supplementary file 1 — Supplementary Information. [file 41598_2020_79254_MOESM1_ESM.pdf]

A Real-World Longitudinal Study of Anemia Management in Non-Dialysis-Dependent Chronic Kidney Disease Patients: a Multinational Analysis of CKDopps

Marcelo Barreto Lopes, Charlotte Tu, Jarcy Zee, Murilo Guedes, Ronald L. Pisoni, Bruce M. Robinson, Bryce Foote, Katarina Hedman, Glen James, Antonio Alberto Lopes, Ziad Massy, Helmut Reichel, James Sloand, Sandra Waechter, Michelle M.Y. Wong, Roberto Pecoits-Filho

Supplemental Table 1. Laboratory values and treatment data at treatment initiation among new users, by type of treatment started

	**Overall**		
	Oral Iron	IV Iron	ESA
Patients, N	370	138	258
% on monotherapy	94%	71%	80%
**Laboratory values***			
CKD stage			
Stage 3	25%	13%	9%
Stage 4	59%	59%	50%
Stage 5	16%	29%	41%
eGFR, ml/min/1.73m^2	24.7(10.6)	20.6(9.4)	18.5(8.2)
Hemoglobin, g/dL	11.2(1.5)	10.9(1.5)	10.2(1.4)
Ferritin, ng/mL	90	101	180

	[47,194]	[51,191]	[95,362]
Ferritin <100 ng/mL	57%	49%	26%
TSAT, %	19.1(9.9)	19.3(10.5)	23.2(9.4)
TSAT <20 %	62%	64%	37%
**ESA type**			
Patients with ESA type data, N	17	34	247
Darbepoetin alfa	29%	38%	50%
Epoetin alfa	65%	50%	34%
Epoetin beta – pegylated	6%	15%	16%
